# Consolidating tactical planning and implementation frameworks for integrated vector management in Uganda

**DOI:** 10.1186/s12936-016-1269-7

**Published:** 2016-04-14

**Authors:** Michael Okia, Peter Okui, Myers Lugemwa, John M. Govere, Vincent Katamba, John B. Rwakimari, Betty Mpeka, Emmanuel Chanda

**Affiliations:** Abt Associates, Kampala, Uganda; National Malaria Control Programme, Ministry of Health, Kampala, Uganda; Nelspruit, Mpumalanga South Africa; Kamwala South, Lusaka, Zambia

**Keywords:** Malaria vector control, Vector borne disease control, Vector control needs assessment, Integrated vector management, Insecticide resistance management, Vector surveillance, Sustainability, Uganda

## Abstract

**Background:**

Integrated vector management (IVM) is the recommended approach for controlling some vector-borne diseases (VBD). In the face of current challenges to disease vector control, IVM is vital to achieve national targets set for VBD control. Though global efforts, especially for combating malaria, now focus on elimination and eradication, IVM remains useful for Uganda which is principally still in the control phase of the malaria continuum. This paper outlines the processes undertaken to consolidate tactical planning and implementation frameworks for IVM in Uganda.

**Case description:**

The Uganda National Malaria Control Programme with its efforts to implement an IVM approach to vector control was the ‘case’ for this study. Integrated management of malaria vectors in Uganda remained an underdeveloped component of malaria control policy. In 2012, knowledge and perceptions of malaria vector control policy and IVM were assessed, and recommendations for a specific IVM policy were made. In 2014, a thorough vector control needs assessment (VCNA) was conducted according to WHO recommendations. The findings of the VCNA informed the development of the national IVM strategic guidelines. Information sources for this study included all available data and accessible archived documentary records on VBD control in Uganda. The literature was reviewed and adapted to the local context and translated into the consolidated tactical framework.

**Discussion:**

WHO recommends implementation of IVM as the main strategy to vector control and has encouraged member states to adopt the approach. However, many VBD-endemic countries lack IVM policy frameworks to guide implementation of the approach. In Uganda most VBD coexists and could be managed more effectively if done in tandem. In order to successfully control malaria and other VBD and move towards their elimination, the country needs to scale up proven and effective vector control interventions and also learn from the experience of other countries. The IVM strategy is important in consolidating inter-sectoral collaboration and coordination and providing the tactical direction for effective deployment of vector control interventions along the five key elements of the approach and to align them with contemporary epidemiology of VBD in the country.

**Conclusions:**

Uganda has successfully established an evidence-based IVM approach and consolidated strategic planning and operational frameworks for VBD control. However, operating implementation arrangements as outlined in the national strategic guidelines for IVM and managing insecticide resistance, as well as improving vector surveillance, are imperative. In addition, strengthened information, education and communication/behaviour change and communication, collaboration and coordination will be crucial in scaling up and using vector control interventions.

## Background

A variety of vector-borne diseases (VBDs) often coexist in the same environment and impose a heavy burden on human populations, particularly in developing countries in tropical and sub-tropical zones [1]. Besides their adverse health effects on humans, VBDs are a significant impediment to socio-economic development. Until now, the control of some VBDs has primarily relied on vertical vector control programmes. The failure to effectively reduce the burden of VBD is due to multiple factors: human, technical (including insecticidal and drug resistance), operational, ecological, economic, and others [2].When well planned and effectively targeted, vector control is an important component of the prevention and management of these diseases. Vector control reduces or interrupts VBD transmission by reducing the vector density or abundance, reducing longevity, and preventing human–vector contact [1]. Therefore, the need for increased access to effective transmission-reducing interventions in areas that are at risk of VBDs cannot be overemphasized.

Vector control methods have evolved from rudimentary environmental sanitation to the present integrated vector management (IVM) approach. The re-emergence of diseases and problems triggered by excessive dependence on insecticides led to the integrated vector control (IVC) strategy described as the “utilization of all appropriate, safe and compatible means of control to bring about an effective degree of vector suppression in a cost-effective manner” [3]. Selective vector control (SVC) evolved the “application of targeted, site-specific and cost-effective activities to reduce malaria morbidity and mortality” [4]. Then followed the concept of comprehensive vector control (CVC) referred to as “control of the vectors of two or more co-prevalent diseases through a unified managerial structure using similar or different vector control methods” [5].

Presently, IVM, defined as “a rational decision-making process for optimal use of resources for vector control” is the recommended approach for vector control [6]. It uses sound principles of management and allows full consideration of the determinants of disease transmission and control. IVM characteristic features include: selection of methods based on knowledge of local vector biology, disease transmission and morbidity; utilization of a range of interventions, often in combination and synergistically; collaboration within the health sector, researchers and with other public and private sectors that have an impact on vector breeding; engagement with local communities and other stakeholders; a public health regulatory and legislative framework; rational use of insecticides; and, good management practices [1]. An IVM strategy takes into account the available health infrastructure and resources, employs a multi-disease approach, and integrates all available and effective interventions, whether chemical, biological, or environmental, involves other sectors and communities, and aims to strengthen vector control management systems [1]. However, introduction of the IVM approach should be preceded by a thorough vector control needs assessment (VCNA) [7].

In Uganda, current approaches to controlling the various VBD work is in near isolation from each other despite the fact that in some districts, opportunities exist for optimal control and management of two or more VBDs by pooling available resources at district and community levels, taking into account health sector reforms wherever possible. In pursuit of integrated VBD control, the Uganda Malaria Reduction Strategic Plan (2014–2020) incorporates principals of IVM [8]. Uganda conducted a VCNA to determine existing gaps in policies, strategies, legislation, and capacity for proper implementation of vector control. The findings of the VCNA informed development of the national IVM strategic guidelines [9]. This paper outlines the processes undertaken to consolidate tactical planning and implementation frameworks for IVM in Uganda.

## Case presentation

The Uganda National Malaria Control Programme (NMCP) with its efforts to implement an IVM approach to vector control was the ‘case’ for this study. Integrated management of malaria vectors in Uganda remained an underdeveloped component of malaria control policy. In 2012, knowledge and perceptions in relation to current malaria vector control policy and IVM were assessed in Uganda, and recommendations for consideration during the development of a specific IVM policy were made. In 2013, an IVM guideline was drafted but focused on malaria and lacked inherent IVM characteristics and components. In pursuit of integrated VBD control, the National Malaria Reduction Strategic Plan (2014–2020) incorporating an IVM approach for vector control was developed and necessitated the updating of the IVM guideline [8]. A VCNA was conducted according to WHO recommendations [7]. The findings of the VCNA informed the development of the IVM strategic guidelines.

Information sources for this study included all available data and accessible archived documentary records on VBD control in Uganda. A methodical review of published and unpublished documents was conducted via a systematic literature search of online electronic databases: Google Scholar [10], PubMed [11] and WHO [12] using a combination of search terms: (1) malaria AND IVM; (2) NTDs AND IVM; (3) NTDs AND vector control; (4) Uganda; (1) and (4); (2) and (4); and, (3) and 4); vector control, epidemiology, malaria, human African trypanosomiasis, leishmaniasis, onchocerciasis, lymphatic filariasis, plague, trachoma, schistosomiasis, tungiasis and arboviruses including’ dengue fever, zika, chikungunya and yellow fever. Additional non-peer-reviewed documents in the Ministry of Health (MoH), such as annual reports and guidelines for vector control, were examined for information related to the subject. The literature was reviewed and applicable findings and key concepts from Uganda and other countries were discussed, adapted to the local context and translated into the consolidated tactical framework.

### The rationale for integrated vector management

While various global strategies have been developed to combat VBDs with renewed emphasis on vector control, malaria still remain the major cause of morbidity and mortality in Uganda [8]. The VCNA revealed several key factors that undermine the effectiveness of vector control in the country: inadequate capacity for evidence-based decision-making to guide vector control strategies at national, regional, district, and community levels, thereby resulting in sub-optimal choice or improper timing of interventions and subsequent waste of valuable resources; vector control efforts focus on a single disease and are not fully integrated into health systems, raising concern about their sustainability; the patterns of most of the VBDs, including malaria, are affected by climate change, environmental degradation and urbanization, pointing to the need for an adaptive management approach to vector control based on local evidence; other sectors such as agriculture, industrial works and construction including communities are often not well informed and involved in vector control, resulting in limited awareness of the consequences of their actions on the incidence of VBDs; long-lasting insecticidal nets (LLINs) and indoor residual spraying (IRS) are threatened by the development of resistance and could undermine effective vector control efforts [9]. The Stockholm Convention on Persistent Organic Pollutants (POPs) [13] and World Health Assembly resolution WHA50.13 [14] call on countries to design sustainable strategies for vector control. These opportunities, coupled with the presence of arboviral diseases also transmitted by mosquitoes, substantiated the need for an IVM approach to vector control in Uganda and the development of guidelines that apply, in principle, to all VBDs but focus specifically on malaria control in the country. The IVM strategic guideline would require regular adaptation to changes in local eco-epidemiological or socio-economic conditions.

### The scale of the vector-borne disease problem

Uganda is located along the eastern African Rift Valley within the Nile basin. It borders Kenya in the east, South Sudan in the north, the Democratic Republic of the Congo in the west, Rwanda in the southwest, and Tanzania in the south. Its topography varies, ranging from high altitude areas, including the Rwenzori Mountains (5100 m) to the low-lying Sudanese Plain in the north. The country has a tropical climate with mean annual temperatures of 16 °C in the southwest; 25 °C in the centre, east, and northwest; and close to 30 °C in the northeast with two peaks of rainfall from March to May and from September to December each year. Uganda has an area of about 241,039 sq km divided into 112 decentralized districts. Service delivery is the responsibility of the districts and their lower local governments. The estimated population of Uganda is 36.6 million people. The proportion of urban area dwellers has increased from 6.6 % in 1969 to 15.6 % in 2011 [15].

Uganda’s equatorial temperature, rainfall and relative humidity provide a conducive environment for malaria and other VBD vectors to thrive (Table 1). However, as malaria is the pathfinder for IVM in Uganda, VBDs targeted initially are those that are amenable to malaria vector control interventions. These include malaria, lymphatic filariasis, plague, visceral leishmaniasis, sleeping sickness, trachoma and arboviruses. Uganda has the third highest number of annual deaths from malaria in Africa and the highest reported malaria transmission intensities in the world [16]. *Anopheles gambiae* s.s., *Anopheles arabiensis* and *Anopheles funestus* are the main malaria vectors. *A. gambiae* s.s. is the dominant vector species in most locations. While these vectors coexist, effective IRS and LLIN interventions have changed their distribution and composition profile in Uganda. Reduced prevalence of *An. funestus and An. gambiae* s.s. and a dominance of *An. arabiensis* has been reported from IRS districts [17]. Secondary vectors include *Anopheles nili*, *Anopheles moucheti*, *Anopheles obscurus* and *Anopheles bwambae* [18]. All the four species of malaria parasites exist in Uganda: *Plasmodium falciparum* (95 %), *Plasmodium malariae* (2 %), *Plasmodium vivax* (2 %), and *Plasmodium ovale* (1 %).The whole population of Uganda is at risk of malaria [19]. Over 90 % of the population resides in areas of high and stable malaria transmission with the remainder in low and unstable malaria transmission areas [8].

**Table 1:** Distribution of vector-borne diseases in Uganda

Disease	Vectors	Population at risk	Areas affected by the disease	No. people infected/annual incidence
Malaria	*Anopheles* mosquitoes	Entire population of Uganda	95 % of country is endemic, 5 % is epidemic	About 60 million fever cases treated annually in government and private facilities 5 % in Kampala to 63 % in mid-northern region
Schistosomiasis	Freshwater snails	5.7 million	63 districts (along large water bodies: lakes, rivers, irrigated areas, etc.)	4 million Prevalence 11–91 %
Lymphatic filariasis (elephantiasis, hydrocele)	*Anopheles*, *Culex* mosquitoes	14.5 million	54 districts (east, north, Bundibugyo)	Prevalence 0.5 (western Uganda)—greater than 40 % (northeastern Uganda)
Onchocerciasis (river blindness)	*Simulium* (blackflies)	4.3 million	37 districts (north, west, east) Eliminated from Mt Elgon focus (Bududa, Mbale, Manafwa, Sironko districts), Itwara focus (Kabarole, Kyenjojo districts), Wambabya focus (Hoima district), Kashoya focus (Kamwenge, Ibanda, Bushenyi districts) Mpamba-Nkusi focus (Kibaale district), Maracha-Terego focus (Arua, Yumbe, Maracha districts), Maramagambo focus (Rukungiri,Bushenyi districts),Wadelai focus (Nebbi district) by 2013	1.4 million infected (this number has declined due to elimination in various foci) Prevalence ten-nearly 100 %
Sleeping sickness	*Glossina* (tsetse flies)	13.6 million	47 districts *Trypanosoma bruceirhondesiense*: 35 districts (southeast and east) *Trypanosoma bruceigambiense*: 12 districts (northwest)	2572 cases reported 2001–2003
Plague	Rat fleas	1.3 million	Zombo district (9/10 sub-counties except Phaida); Arua district (two sub-counties) and Nebbi district are affected	Recent outbreaks 2006: 127 cases (11 fatal) in Arua and Nebbi districts 2007: 179 cases (nine fatal) in Masindi, 121 cases (ten fatal) in Arua, 39 cases (nine fatal) in Nebbi districts 2008: 68 deaths in Arua and Nebbi districts
Leshmaniasis	Sandflies	1.2 million	7 districts in northeast (Karamja region: Amudat, Nakapiripirit, Moroto, Kaabong, Kotido, Napak, Abim districts)	408 cases reported in 2003
Trachoma	Houseflies (*Musca* spp.)	10.8 million	41 districts in east, north	Not known
Tungiasis	Jigger fleas	33.1 million	112 districts (countrywide) but mainly in the Busoga region	Not known

The threat for emerging and re-emerging vector borne diseases i.e. dengue, chikungunya and lymphatic filariasis is increasing in East Africa. The VCNA revealed the presence of multiple VBDs with divergent endemicities and spatial distribution in Uganda, including: Schistosomiasis vectored by *Bolinus* and *Biomphalaria* spp. snails; dengue fever primarily transmitted by *Aedes aegypti* and *Aedes albopictus*; human African trypanosomiasis vectored by *Glossina fuscipes*, *Glossina tachinoides*, *Glossina pallidipes* and *Glossina morsitans* species of tsetse flies; Leishmaniasis (kala-azar) vectored by *Phlebotomus orientalis*, *Phlebotomus martini and Phlebotomus celiae* species of Sand flies; Onchocerciasis (River Blindness) vectored by *Simulium damnosum* black flies; lymphatic filariasis transmitted by *A. gambiae*, *An. arabiensis* and *An. funestu* and *Culex quinquefasciatus*; chikungunya and zika vectored by *Ae. albopictus* and *Aedes aegypti*; Trachoma vectored by *Musica sorbens* bazaar flies; plague vectored by *Xenopsilla* spp. Rat fleas; Tungiasis transmitted by *Xenopsilla* spp. Jigger fleas and Rift valley fever transmitted by *Aedes* spp. mosquitoes. Meanwhile, malaria targeting interventions such as IRS and LLINS also have a significant effect of on other vectors of VBDs i.e. dengue, chikungunya, zika, lymphatic filariasis, yellow fever and leishmaniasis.

### Vector control interventions in Uganda

The large communal-scaleuse of LLINs and IRS, supported by intensive social mobilization and behaviour change communication (BCC), are key interventions to prevent malaria and other VBDs in Uganda. The country has adopted a policy of universal coverage (one net per two people) with LLINs to protect all people from malaria and other VBDs with over 21 million nets distributed via mass campaigns. Since 2009, two spray rounds per year have been conducted in ten districts covering over 500,000 structures, achieving >90 % coverage of targeted structures and protecting over 2.6 million representing >90 % of the targeted population. In the northern and eastern regions with holo-endemic malaria transmission, LLINs and IRS are combined for the same households [19]. While there are currently no meaningful larval source management (LSM) activities at programme level, the MoH plans to implement LSM in the context of IVM in urban or peri-urban sites and arid areas of Uganda. Live-bait technology, application of insecticides on domesticated animals, especially cattle to control tick borne diseases is practiced by local communities in Greater Mbarara district. Community sensitization on the benefits of this approach to controlling several VBDs including sleeping sickness, malaria, lymphatic filariasis, and trachoma, and the expansion of these efforts into other cattle-keeping communities is required. Tsetse traps and screens are used in tsetse-infested areas such as eastern and northern Uganda and can also be used around zero-grazed cattle, to help reduce domestic animal nuisance pests, including stomoxys and tabanid flies, and to some extent malaria vectors. These devices also help reduce eye-seeking flies, thereby helping to control trachoma [20].

### Challenges for vector-borne disease control

In Uganda major challenges to malaria control include very high malaria transmission intensity, low coverage of proven malaria control interventions, inadequate health care resources, a weak health system, inadequate understanding of malaria epidemiology and the impact of control interventions [16]. There has been a growing problem of insecticide resistance in disease vectors to organochloride, dichloro-diphenyl-trichloroethane (DDT), pyrethroids and to a lesser extent carbamates, particularly in *An. gambiae* s.l. and *An. funestus* [21, 22]. Integrated management of malaria vectors remains an underdeveloped component of malaria control policy in Uganda [23]. Most development partners have neglected health system strengthening [24]. Therefore, concerted efforts and strong political commitment that translates into adequate financial allocation for vector control, including focused research and inter-country cooperation and exchange to share best practices are urgently needed to radically scale up cost-effective interventions. Further challenges include inadequate social mobilization and sensitization of target communities due to limited resources for requisite activities. Integrated control would require strengthening of research, including studies of the efficacy and cost-effectiveness of integrated management of VBDs, knowledge, attitude and practice, community meetings, and development of and harmonization of information, education and communication (IEC) messages and their effective delivery of materials [25].

### Consolidating the strategic framework for vector control

The current approaches to disease vector control in Uganda work in near isolation from each other, despite the existence of opportunities for optimal control and management of two or more VBDs in some districts [20]. To the extent possible, the available vector control tools need to be integrated in Uganda by adopting and implementing the IVM strategy. In 2013, an IVM guideline was drafted but focused on malaria and lacked inherent IVM characteristics and components. In pursuit of integrated VBD control, the National Malaria Reduction Strategic Plan (2014–2020) incorporating an IVM approach for vector control was developed and necessitated the updating of the IVM guideline [8]. An IVM strategy takes into account the available health infrastructure and resources, employs a multi-disease approach, and integrates all available and effective interventions, whether chemical, biological or environmental, involves other sectors and communities, and aims to strengthen vector control management systems [1]. IVM characteristic features include: selection of methods based on knowledge of local vector biology, disease transmission and morbidity; utilization of a range of interventions, often in combination and synergistically; collaboration within the health sector, researchers and with other public and private sectors that have an impact on vector breeding; engagement with local communities and other stakeholders; a public health regulatory and legislative framework; rational use of insecticides; and, good management practices [1].

### Vector control needs assessment

Introduction and implementation of IVM requires a comprehensive situation analysis, and an in-depth assessment of challenges and specific vector management needs. Prior to developing an IVM strategy, a VCNA was conducted to assess existing gaps in policies, strategies, legislation, and capacity for the implementation of vector control in the context of IVM.A modified World Health Organization VCNA tool [7] was used to collect data from key informants from the NMCP, 17 districts and institutions that included the Vector Control Division (VCD), Uganda National Bureau of Statistics, Uganda Revenue Authority, National Environmental Management Authority, National Drug Authority, and Agricultural Chemical Board of the Ministry of Agriculture, Animal Industry and Fisheries. The findings of the VCNA established that there is goodwill by both government and international organizations to support IRS and LLINs, which provide a platform for resource mobilization. While implementation of LSM is rudimentary, there is local capacity to effectively implement IRS and LLINs using a partnership model that creates room for sustainability of the IVM interventions. To manage insecticide resistance, carbamates (bendiocarb) or organophosphate (pirimiphos-methyl CS) are used for IRS [26]. The Agricultural Chemicals (Control) Act, 2006 exists with attendant regulations and guidelines to handle all agricultural chemicals along the value chain [9]. However, vector control in Uganda has been characterized by many constraints. Table 2 presents the synopsis of key challenges/issues and recommendations for improving vector-borne disease control in Uganda.

**Table 2:** Challenges and risks encountered in vector control and recommendations for improvement in Uganda

Challenges and risks encountered	Recommendations for improvement
There are no vector control guidelines to enable districts to effectively implement the IVM strategy	Develop, print, and disseminate IVM guidelines, training, and information, education and communication materials to districts and all IVM implementing partners
There is no national IVM steering committee or IVM focal person to provide technical guidance for IVM and to link up with various sector ministries/organizations	Establish a national IVM steering committee to coordinate IVM implementation with the MoH acting as the committee secretariat
There is no appropriately qualified IVM focal point person in the MoH	Appoint a senior officer with a minimum qualification of MSc in Medical Entomology as the IVM focal person in the MoH
There is no functional inter-sectoral collaboration, either within or outside the MoH	Establish inter-sectoral collaboration within and outside the MoH for effective IVM implementation
Vector control officers at both national and district levels lack the necessary resources, infrastructure, and logistics to implement the IVM	Have the MoH provide the necessary resources, infrastructure, and logistics for IVM implementation
There are no human resource development plans and career development opportunities for vector control staff at either the national or district level	Put in place career development and promotional opportunities for vector control staff
There is no legal framework to manage public health pesticides and environment	Establish a legal framework to manage public health pesticides
Community involvement in vector control implementation is minimal	Mobilize and empower communities to actively participate in IVM activities
The LLIN distribution programmes are not supported by comprehensive IEC/BCC campaigns to promote use	The MoH and partners should strengthen IEC/BCC to enhance uptake of interventions
There is limited capacity at the National Drug Authority, National Environmental Management Authority and Uganda National Bureau of Statistics to execute their mandate of monitoring the use and quality of public health pesticides	Strengthen monitoring and quality control for LLINs by the Uganda National Bureau of Standards (UNBS) and National Drug Authority (NDA)

### Integrated vector management

In 2014, Uganda developed the IVM strategic guideline to provide a common operational framework for all vector control stakeholders for planning and implementing VBD control according to IVM characteristics and principles [20]. The guideline seeks to reorient vector control interventions in the context of emerging insecticide resistance, the need for inter-sectoral collaboration and targeted evidence-based vector control. The development of the guideline was all-inclusive and participatory, involving all key stakeholders, including civil society, academia/research, malaria development partners, and the private corporate sector. The document will be regularly adapted to changes in local eco-epidemiological or socio-economic conditions. The selection criteria for malaria vector control methods include effectiveness, affordability, safety, sustainability, risk for resistance, community acceptance, etc. (Tables 3, 4).

**Table 3:** Integrated vector management interventions to reduce vectorial capacity

Objective	Action	Method
Reduce vector abundance	Reduce the number of sites where vector larvae grow	Environmental management
	Reduce number of larvae or prevent insects from reaching adult stage	Larvivorous fish, biolarvicides, insect growth regulators, and other parasites, chemical larvicides
	Kill adult insects when they rest on sprayed surfaces	Indoor residual spraying (IRS)
	Kill insects as they alight on treated surfaces, repel them or inhibit them from feeding/biting	Insecticide-treated materials such as mosquito nets, curtains and *chaddars* (cloth sheets), indoor durable wall linings
	Reduce the population of adult insects	Space spraying in urban areas.
Reduction of vector survival and longevity	Reduce life of the insect before it reaches infective age	IRS and insecticide-treated materials
Reduction of human vector contact	Reduce opportunities for insects to enter the house	House improvements such as screens with insecticide-treated materials and wiremesh
	Repel insects before they bite	Repellents: coils, mats, lotions, creams, vaporizers, etc.

**Table 4:** Methods for controlling vector-borne diseases and their respective effects on vector abundance, survival and human contact

Disease/pest	Main vector	Chemical control					Biological control		Environmental management	Legislation
		IRS	LLINs	Repellents	Larviciding	Space spraying	Larvivorous fish	Biolarvicides		By laws, regulation
	Effects on elements of vectorial capacity	1, 2, 3	1, 2, 3	3	1	1, 3	1	1	1	Supportive regulation
Malaria	*Anopheles* spp.	+	+	+	+	+	+	+	+	+
Leishmaniasis	*Phlebotomus* spp.	+	+	**−**	**−**	**−**	**−**		+	+
Filariasis	*Culex* spp., *Mansonia* spp., *Anopheles* spp.	+	+	+	+	−	+	+	+	+
Houseflies	*Musca domestica*	+	**−**	**−**	**−**	+	**−**	**−**	**−**	+
Cockroaches	*Periplanata* spp.	+	**−**	**−**	−	**−**	**−**	**−**	+	**−**
Bedbugs	*Cimex* spp.	+	+	**−**	−	**−**	**−**	**−**	+	**−**
Fleas	*Xenopsylla* spp.	+	+	**−**	−	**−**	**−**	**−**	+	+
Rodents	*Various* spp.	**−**	**−**	**−**	−	**−**	**−**	**−**	+	**−**
Snails	*Biomphalaria* spp., *Bulinus* spp.	**−**	**−**	+	+	**−**	+	+	+	+

1 = Reduce abundance, 2 = reduce survival/longevity, 3 = reduce human-vector/pest contact, + = effect, − = no effect

The mechanisms for implementation of the IVM strategic guidelines will be a joint effort by all partners and stakeholders at all levels of society through the public health system (Table 5). The MoH via the NMCP and VCD will coordinate efforts to control malaria and other VBDs using the platform of Interagency Coordination Committee for Malaria (ICCM), which is chaired by the MoH. For effective inter-sectoral collaboration, an inter-sectoral steering committee on IVM will function as an inter-ministerial governing body with a responsibility to facilitate harmonization of policies and institutional arrangements and to provide strategic direction and coordination for research and implementation of IVM. A nominated focal person for IVM will coordinate and manage networking among national partners and ensure implementation of the recommendations of the Committee.

**Table 5:** Anticipated roles of various sectors in integrated vector management implementation

Sector/agency	Roles
NMCP	Resource mobilization for IVM. Setting strategic directions and conducting overall evaluation; advising on policy and institutional arrangements; conducting epidemiological and vector assessment, stratification; supervising decentralized planning and implementation; supervising decentralized monitoring and evaluation; supervising decentralized organization and management; preparing curricula and training trainers; ensuring preparedness to coordinate emergency response; advising on research priorities
Districts and villages	Establishing inter-sectoral partnerships and networking; planning and implementing local IVM strategy; implementing health interventions; monitoring and evaluating; organizing and managing; undertaking local vector surveillance; providing training, education and awareness raising
VCD	Support the NMCP in planning, implementing, supervision, monitoring and evaluation of IVM implementation of interventions. Monitor impact of malaria control interventions on other VBDs. Support the NMCP in conducting capacity building for malaria vector control; monitor insecticide resistance in vectors to control commodities. Monitor the impact of application of chemicals on domestic animals in the control of tsetse flies on malaria vectors as well as on sleeping sickness
Agriculture	Ensure that famers implement IVM, popularizing the concept of dry-wet irrigation through extension education, management of agricultural pesticides. Coordinate and monitor the impact of the application of chemicals onto domestic animals on the control of tsetse flies and ticks and tsetse- and tick-borne diseases. Ensure the safe use and application of agricultural chemicals to protect human, and animal health and environment
Water resources development	Maintenance of canal system, intermittent irrigation, design modifications and lining of canals, weeding for proper flow, creating small check-dams away from human settlements, health impact assessment
Water supply	Repair of leakages to prevent pooling, restoration of taps, diversion of wastewater to pond/pit, staggering of water supply, mosquito-proofing of water harvesting devices, repair of sluice valves
Local governments	Implement, supervise and monitor IVM implementation. Implement the Kampala Declaration on Sanitation
Road and building sector	Proper planning as per bylaws, merging pits by breaking bunds, excavations in line with natural slope/gradient, making way for water to flow into natural depression/pond/river, follow-up actions after excavations, maintaining storm water roadside drains
Urban development	Implementation of building bylaws, improved designing to avoid undue water lodging, building use permission after clearance of health dept; safe rainwater
National Environment Management Authority	Ensure Environmental Impact Assessments are done before embarking on projects. Provide guidelines in the manufacture and handling of public health insecticides, storage and disposal including safety measures to prevent human and environment contamination
National Drug Authority	Develop legislation for the manufacture, sale, importation, storage, transportation, and use of public health insecticides. Develop and disseminate specific operational guidelines on the management of pesticides for public health. Ensure the quality of public health pesticides imported into the country. Ensure the quality and quantity of insecticide in LLINs. Monitor quality of public health pesticides, equipment, etc. in the open market
Uganda National Bureau of Standards	Ensure the quality of LLINs imported into the country conform to international standards (WHOPES recommendations)
CBO, FBO and CSOs	Participate in implementation of some IVM interventions, e.g., distribution of LLINs and conducting IRS, conduct advocacy, social mobilization and BCC and community sensitization. Support active community participation in malaria control and prevention activities
Private sector	Manufacture and procure quality IVM logistics (equipment and chemicals), participate in implementation of some IVM interventions e.g., distribution of LLINs and conducting IRS
Communities	Participate in implementation of some IVM interventions, e.g., distribution of LLINs, conducting IRS and larval mosquito control, proper use and care of LLINs. Apply chemicals on domestic animals for controlling tsetse flies, ticks and malaria vectors for controlling sleeping sickness, tick-borne diseases and malaria
USAID, DFID, ADB, GFATM, World Bank, UNICEF	Provide the resources for IVM interventions, monitor and evaluate IVM interventions
WHO	Provide the resources for IVM interventions, monitor and evaluate IVM interventions, provide IVM guidelines and training and IEC materials
Research institutions	Conduct research on vectors and VBD and impact of IVM interventions on VBD
Universities	Conduct training of vector control staff, conduct research on disease vectors and VBD and impact of IVM interventions on VBD
Media	Highlight the public health and socio-economic impact of malaria and other VBDs. Raise the profile of and demand for IVM interventions through targeted, well-designed advocacy and communication campaigns and activities. Advocate for increased resources allocated for malaria control. Promote IVM as a method for vector control

## Discussion

WHO recommends implementation of IVM as the main strategy to vector control and encourages member states to adopt the approach. However, many VBD-endemic countries lack IVM policy frameworks to guide implementation. By 2010, only 62 % of 113 countries had developed policies on IVM and implemented the strategy [27]. While most VBDs coexist in Uganda and could be managed more effectively if done in tandem, integrated management of disease vectors remains underdeveloped. Uganda assessed knowledge and perceptions in relation to current malaria vector control policy and IVM, and made recommendations for consideration during future development of a specific IVM policy [8]. By 2014, Uganda successfully developed evidence-based national strategic guidelines for IVM founded on thorough VCNA [17].

Uganda is among the countries with the highest malaria transmission intensities worldwide [16]. In order to successfully control malaria and other VBDs and move towards their elimination, the country needs to learn from the experience of other countries, and scale up proven high-impact vector control interventions. Some endemic countries have put vector control high on the political agenda using the IVM strategy as a platform [28]. Vector control has been implemented and sustained with consistent national government funding supplemented by partner support [29, 30]. LLINs and IRS are deployed as main-thrust malaria vector control tools supplemented with LSM in accordance with and strict adherence to a set of eligibility criteria [29, 31]. In Zambia, IVM has facilitated strong partner collaboration and has helped leverage additional resources for vector control [31]. Namibia and Eritrea have recently developed costed IVM strategies for advocacy and leverage of resources for evidence-based vector control [28, 29]. With the escalating problem of insecticide resistance in malaria vectors [32, 33], insecticide resistance management (IRM) and improving vector surveillance in Uganda will be crucial. Equally, strengthening IEC/BCC and consolidating inter-sectoral collaboration and coordination will be needed to improve uptake and support for the implemented tools.

Strong entomological teams at national and local levels are crucial to coordinate routine monitoring of resistance, data analysis and interpretation to inform policy decisions, translate policies and guidance into action at ground level [34]. Although current resistance data show that organophosphates are the only technically sound options for IRS in Uganda, a rational IRM approach is required to guide evidence-based decisions regarding insecticide choices for vector control. However, switching to strategic IRM demands for development of monitoring and management plans based on a meticulous review of information on insecticides registered for public health and agricultural use, knowledge of main vector species and their resistance profiles, efficient data management and mapping capacity, current vector control interventions, evidence and knowledge gaps including constraints and mitigating measures [35]. The plan should incorporate an implementation framework comprising a multidisciplinary national IRM decision-making body, the establishment of multiple sentinel sites for routine resistance surveillance and monitoring and collection of data annually, including interpretation of test results and policy implications, human resources, procurement and supplies, regulatory requirements and procedures, budget and potential sources of funding [35]. Zambia, Eritrea, South Africa, Mozambique, and Namibia have developed country-specific IRM plans to prevent development and spread of insecticide resistance and have trained local staff in entomological surveillance and resistance monitoring.

Mapping of vector species and their resistance profiles across the country would facilitate deployment of cost-effective vector control. As such, vector surveillance should be an integral aspect of the IVM strategy implementation to: (1) provide evidence for decision-making in IRS; (2) evaluate a programme’s impact on vector populations; and, (3) monitor and evaluate IRS where the surveillance sites are located in or near implementation settings. These investigations will provide data on temporal and spatial vector species composition, density, feeding and resting behaviour, infectivity rate and longevity, susceptibility to insecticides, and quality and residual effect of insecticides [36]. Therefore, Uganda should develop country-specific vector surveillance guidelines to include: entomological field and laboratory techniques, WHO contact and susceptibility bioassays, mosquito rearing in insectaria, organization of entomological teams, geographical information systems (GIS), and supervision of entomological teams and operations [36]. Entomological investigations should be conducted regularly at fixed locations to: (1) reduce natural variation, costs and labour intensity; (2) increase the usefulness of timely collected data in decision-making; and, (3) optimize the use of available resources. Eritrea and Namibia have elaborated vector surveillance guidelines to facilitate entomological monitoring by the regional levels [30].

The mandate of informing, educating and mobilizing the communities on VBD control operations in Uganda rests with the MoH via the NMCP and VCD. This requires unremitting and well-coordinated IEC/BCC to promote knowledge, awareness and compliance and ownership vector control tools. Equally, strategic and effective advocacy to mobilize local public and private support will be vital for the sustainability of the IRS programme. Therefore, the NMCP should: (1) coordinate the development of all IEC and advocacy material for vector control and ensure the harmonization of its content in collaboration with partners; and, (2) advocate for increased and continued political, financial and technical support and mobilize all stakeholders and partners for vector control. For effective collaboration to link various stakeholders at national level, an inter-sectoral steering committee on IVM will function as an inter-ministerial governing body with a responsibility to facilitate harmonization of policies and institutional arrangements, interaction between Ministries/Departments and to provide strategic direction and coordination for research, planning and implementation of IVM. This committee will also serve as a platform for reconciling competing interests between Ministries/Departments at the national level or navigating IVM when partners or competing interests disagree with the process or plan.

The IVM strategy is important in consolidating inter-sectoral collaboration and coordination and providing the tactical direction for effective deployment of vector control interventions along the five key elements of the approach and to align them with changing epidemiology of VBD in Uganda. It outlines strategic interventions and activities, IRM, monitoring and evaluation and operational research, programme management, budget and funding, an implementation plan, and the performance framework [20]. The IVM strategy is expected to reduce the risk of transmission, reduce disease burden, improve the cost effectiveness of vector control operations, improve ecological soundness and be sustainable. Cooperation between the health and other sectors needs strengthening and funding for vector control increased in order to develop and effectively implement an appropriate IVM policy. Zambia and Zimbabwe have established strong external links with international research institutions to further build local entomological capacity [31]. Continuous engagement of communities by government as well as monitoring and evaluation of vector control programmes will be crucial for sustaining IVM in Uganda. In fact, requisite resources for vector control could be mobilized by strengthening advocacy and collaboration based on the IVM strategy.

## Conclusions

Uganda has successfully consolidated strategic planning and operational frameworks for VBD control by establishing an evidence-based IVM approach. This will help the country to expedite their efforts towards achieving VBD elimination/eradication, particularly malaria. The country should put into operational implementation arrangements as outlined in the IVM strategic guidelines. IRM should be implemented and vector surveillance improved. In addition, strengthened IEC/BCC and consolidating inter-sectoral collaboration and coordination will be crucial for scaling up and utilization of vector control interventions in the country.

## Abbreviations

BCC: behaviour change communication
CBO: Community Based Organization
CSO: Civil Society Organization
CVC: comprehensive vector control
DDT: dichloro-diphenyl-trichloroethane
FBO: Faith Based Organizations
GIS: geographical information systems
GPIRM: Global Plan for Insecticide Resistance Management
ICCM: Interagency Coordination Committee for Malaria
IEC: information, education and communication
IVC: integrated vector control
IRS: indoor residual spraying
IRM: insecticide resistance management
IVM: integrated vector management
IRMTWG: Insecticide Resistance Management Technical Working Group
LLINs: long-lasting insecticidal nets
LSM: larval source management
NMCP: National Malaria Control Programme
NTDs: neglected tropical diseases
MoH: Ministry of Health
POPs: persistent organic pollutants
SVC: selective vector control
VBD: vector-borne disease
VCNA: vector control needs assessment
VCD: Vector Control Division
WHA: World Health Assembly
WHO: World Health Organization
